# The Mechanism of Bladder Injury in Fetal Rats With Myelomeningocele

**DOI:** 10.3389/fneur.2022.861308

**Published:** 2022-06-09

**Authors:** Ying Liu, Li Chen, Yunli Bi, Jian Shen, Hong Chen, Yujie Ma

**Affiliations:** ^1^Department of Urology, Children's Hospital of Fudan University, Shanghai, China; ^2^Department of Urology, Children's Hospital of Fudan University at Xiamen (Xiamen Children's Hospital), Xiamen, China; ^3^Department of Urology, Children's Hospital of Soochow University, Soochow, China

**Keywords:** myelomeningocele fetal rats, bladder nerve, immunohistochemistry, apoptosis, proliferation, inflammatory, neurogenic bladder

## Abstract

**Background:**

Bladder dysfunction has been implicated as a major cause of progressive renal failure in children with neurogenic bladder. However, its pathogenesis remains unclear. This study aimed to compare the expression of proliferation, apoptosis, and neuromuscular-related proteins during the development of the bladder in myelomeningocele fetal rats, and to explore the characteristics of its abnormal development.

**Methods:**

For the myelomeningocele group, Sprague Dawley pregnant rats were intragastrically injected with retinoic acid on the 10th day of gestation to induce myelomeningocele fetal rats. For the control group, the same amount of olive oil was injected to induce normal fetal rats. Bladders were harvested at embryonic days E16, E18, E20, and E22. Real-time quantitative polymerase chain reaction and western blotting were used to detect the protein levels of proliferating cell nuclear antigen (PCNA), cleaved caspase-3, neuron-specific nuclear-binding protein (NeuN), α-smooth muscle actin (α-SMA), and mRNA at E16–E22; immunohistochemistry was used to detect the expression of cleaved caspase-3 at E22.

**Results:**

The proliferation of bladder tissue cells was inhibited, with suppressed PCNA expression in myelomeningocele bladder tissue compared with that in control tissue at the early stage (E16). Myelomeningocele bladders showed increased tissue apoptosis in the late embryonic stage, with significantly higher cleaved caspase-3 protein expression than in the control bladders at E20 and E22. NeuN protein expression increased along with embryonic stage, although the expression at E20 and E22 was significantly lower in myelomeningocele bladders than in control bladders. α-SMA protein expression in myelomeningocele bladders increased gradually with the progression of pregnancy, although its expression was lower than that for control bladders at E22. Immunohistochemistry showed abundant positive staining for cleaved caspase-3 in the bladder mucosa and muscle layer of myelomeningocele bladders, and the expression of cleaved caspase-3 was significantly higher in myelomeningocele bladders than in control bladders.

**Conclusions:**

Bladder dysfunction in myelomeningocele fetal rats is related to the inhibition of proliferation, promotion of apoptosis, and reduction of bladder nerve and smooth muscle-related protein synthesis.

## Introduction

Neurogenic bladder (NB) is a complex disease and historically is a major cause of morbidity and mortality in children with myelomeningocele (MMC). MMC is a common congenital malformation of the central nervous system in children. Since the 1980s, its global incidence rate has generally declined due to selective abortions enabled by advanced prenatal screening (maternal serum alpha-fetoprotein, ultrasound, and amniocentesis) and mandatory folic acid supplementation during pregnancy (1, 2). However, its incidence in neonates has remained high in China (above 1/1,000 births) for many years (3). Although 75% of children with MMC can grow to the adult stage, they have lifelong disabilities, and clinical MMC with urinary tract dysfunction seriously affects their quality of life and social and work abilities (4).

Although fetal surgery has greatly advanced and the early repair of spinal cord myelomeningocele (the main treatment to avoid further loss of nerve tissue) can improve the clinical prognosis (5, 6), these children still have congenital malfunctions, and improvement in urinary and gastrointestinal function is poor (4, 7). Previous studies found that the structure and function of the bladder in children with MMC are significantly abnormal (8, 9). However, it is unclear whether the abnormal structure and function are the result of a progressive deterioration of nerves during pregnancy or the existence of a developmental malformation of the bladder. Therefore, this study aimed to explore the development of the bladder from embryonic days E16–E22 in fetal rats with retinoic acid-induced spinal myelomeningocele. To elucidate the mechanism of NB, changes in proliferation, apoptosis, and neuromuscular development were evaluated at each time point.

## Materials and Methods

### Establishment of the Animal Model

All animal studies were approved by the Research Ethics Board of the Children's Hospital of Fudan University. Male and female Sprague Dawley (SD) rats were purchased from the Shanghai Shrek Company and raised in the animal room at the Shanghai Medical College of Fudan University (laboratory animal license: SYXK 2014-0029).

An animal model of MMC in fetal rats was developed by intragastric administration of retinoic acid. All-trans retinoic acid (ATRA) was administered to the SD rats on the 10th day of gestation. The rats in the experimental group were administered with 60 mg/kg of ATRA by gavage, while the rats in the olive oil group were administered with 2 mL of olive oil by gavage. At E16, E18, E20, and E22, the fetal rats were removed by laparotomy. The rats with the MMC phenotype in the experimental group were designated as the MMC group, while the rats in the olive oil group were designated as the control group (CRL).

The urachal was found in the lower abdomen, which was then explored down to the bladder. Some bladder tissue was dissociated into the posterior urethra and immersed in 10% paraformaldehyde for pathological analysis, and the other tissue was stored in RNA-free Eppendorf tubes, which were transferred to a refrigerator and maintained at−80°C via liquid nitrogen for subsequent molecular biology experiments. The weight of the bladder was measured and recorded on an electronic scale, and the head-hip length of fetal rats was measured and recorded using a Vernier caliper to evaluate the intrauterine development of MMC fetal rats.

### Immunohistochemistry of Fetal Rat Bladders

Tissue was fixed in 4% paraformaldehyde, dehydrated, soaked in wax, and embedded in wax blocks. Pretreated 5-μm tissue sections were dewaxed, rehydrated, and antigen-repaired under high temperature and pressure. Subsequently, endogenous peroxidase was blocked with 3% serum for 10 min. Thereafter, sections were incubated with cleaved-caspase 3 (1:400, Cell signaling Tech, USA)at 4°C overnight, washed with tris-buffered saline and tween (TBST) buffer thrice, and finally incubated with a poly-HRP anti Ms/Rb/Rt for 30 min (Gene Tech, Shanghai) at room temperature for 2 h. Finally, the sections was washed three times with TBST buffer, followed by staining with DAB(Gene Tech, Shanghai) for 7 min and counterstaining with hematoxylin for 1 min. And then sealed and photographed with a fluorescence microscope.

### Western Blotting

Proteins were extracted from the tissues using ultrasound, and the protein concentration was determined using a bicinchoninic acid Protein Assay kit (Beyotime Biotechnology Co Ltd, Shanghai, China). A 12% sodium dodecyl sulfate polyacrylamide gel electrophoresis gel was used for electrophoresis, and proteins were transferred to a polyvinylidene fluoride membranes (Merck KGaA, Darmstadt, Germany). After blocking the membranes with 5% skimmed milk, the membranes were incubated with the first antibody (Table 1) at 4°C overnight. The membranes was then washed with TBST buffer three times, and incubated with the secondary antibody at room temperature for 2 hours. The membranes was washed again with TBST buffer, and the results were detected using a gel imaging system with the ImageQuant LAS 4000 series device (GE Healthcare Life Sciences, Marlborough, MA, USA) following reaction with an enhanced chemiluminescence solution (Thermo Fisher SCIENTIFIC, USA).

**Table 1:** Antibodies used for western blotting.

**Name**	**Manufacturer**	**Dilution**
Cleaved-caspase 3	Cell signaling tech	1:1000
PCNA	Proteintec	1:2000
NeuN	Cell signaling tech	1:1000
α-SMA	Cell signaling tech	1:1000
HRP-linked goat anti-mouse and rabbit polyclonal antibody	Mai Bio Co	1:2000

*PCNA, proliferating cell nuclear antigen; α-SMA, α-smooth muscle actin; NeuN, neuron-specific nuclear-binding protein*.

### Real-Time Quantitative PCR Detecting System

Trizol reagent (1 mL) was added to RNA-free Eppendorf tubes containing bladder tissue, ground with a grinder for 20 s, followed by resting without grinding for 10 s, until there was no residue at the bottom of the Eppendorf tube. The tubes were then allowed to stand at room temperature for 5 min. After centrifugation at 12,000 rpm at 4°C for 5 min, the supernatant was placed into another Eppendorf tube, 200 μL of chloroform was added, and the centrifuge tube was tightly covered and mixed vigorously for 25 s. The mixture was then allowed to stand at room temperature for 5 min. After centrifugation at 4°C and 12,000 rpm for 15 min, the upper colorless aqueous phase containing RNA was transferred to another Eppendorf tube, 0.5 mL of isopropanol was added, and the tube was placed upside down and mixed well. After centrifugation, samples were left in a refrigerator at 4°C for 15 min. After centrifugation at 4°C and 12,000 rpm for 10 min, transparent precipitates were at the bottom of the tube. The supernatant was discarded and 1 mL of precooled 75% ethanol was added, and the mixture was turned upside down until the precipitate floated in 75% ethanol. After centrifugation at 4°C and 7,500 rpm for 5 min, the supernatant was discarded, dried, and precipitated at room temperature for 1–2 min, and 20–50 μL of RNase-free water was then added to dissolve and precipitate the mixture. The extracted RNA was reverse-transcribed according to the instructions of the PrimeScript RT Reagent Kit (AKALA, Japan). Primers were diluted according to the manufacturer's instructions (Table 2). Real-time quantitative PCR (qPCR) was performed using a 20-μL system: primer I, 0.8 μL; primer II, 0.8 μL; TB Green, 10 μL; and cDNA, 1 μL. Gene expression levels were calculated using the 2 ^−ΔΔCT^ method (10).

**Table 2:** Sequences Used for qPCR.

**Name**	**Sequence**
PCNA-F	AGAGCATGGATTCGTCTCACG
PCNA-R	TGGACATGCTGGTGAGGTTC
Cleaved caspase-3-F	TACCCTGAAATGGGCTTGTGT
Cleaved caspase-3-R	GTTAACACGAGTGAGGATGTG
α-SMA-F	TCTGGTGTGTGACAATGGCT
α-SMA-R	CACGATGGATGGGAAAACAGC
NeuN-F	ACCGCCGTCGCCTATCG
NeuN-R	GGGCTTTTGGAGGTTTGAGA

*PCNA, proliferating cell nuclear antigen; SMA, smooth muscle actin; NeuN, neuron-specific nuclear-binding protein*.

### Statistical Analyses

All numerical data are presented as means and standard deviations (STDs). Significance was defined as *P* < 0.05. All analyses were performed using the IBM SPSS Statistics for Windows, ver. 22 (IBM Corp., Armonk, NY, USA).

### Ethical Considerations

This study was approved by the Institutional Review Board of the Children's Hospital of Fudan University.

## Results

### Alterations Observed in MMC Induced by ATRA

**Figure 1:**
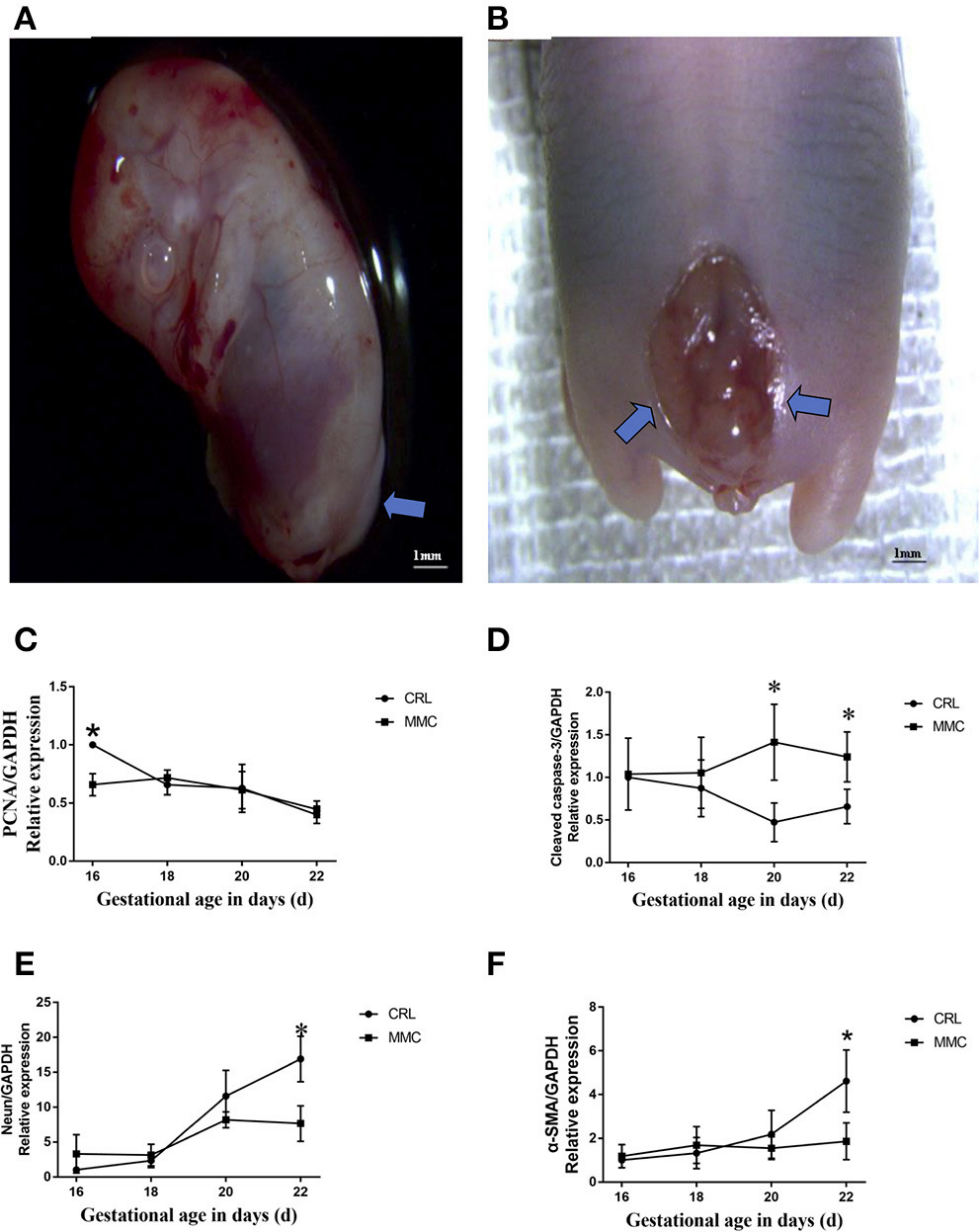
Representative photographs of spinal cord in fetuses with lumbosacral myelomeningocele. **(A)** E16, neural tube closure failed, nerve tissue is exposed dorsally and protrudes above the skin level. **(B)** E20, the myelomeningocele exhibits abrasion and hemorrhage, a thin fibrous membrane covers the defect. **(C–F)** The mRNA expression levels of PCNA **(C)**, cleaved caspase-3 **(D)**, NeuN **(E)**, and α- SMA **(F)** in the MMC and CRL groups changed with the development of the embryos, *n* = 5, ^*^P < 0.05. Data are expressed as means ± STDs. MMC, myelomeningocele; CRL, control; STD, standard deviation; PCNA, proliferating cell nuclear antigen; α-SMA, α-smooth muscle actin; NeuN, neuron-specific nuclear-binding protein.

Among 33 adult female SD rats, 21 were fed retinoic acid during pregnancy; among 182 fetal rats, 66 did not show the MMC phenotype (discarded), and 116 had MMC. The number of fetal rats evaluated at E16, E18, E20, and E22 was 31, 30, 26, and 29, respectively. The defect was mainly concentrated in the lumbosacral segment, with an incidence rate of 63.7%, which was close to the modeling rate reported by the international community (9, 11). On evaluation, we found that in early pregnancy (E16 and E18), the MMC was cystic and protruded from the back skin; in the third trimester of pregnancy (E20 and E22), the MMC showed an extremely flat back, erosion, and degeneration, with a thin fibrous membrane covering the defect (Figures 1A,B).

### Delay in Growth and Development in MMC Fetal Rats

MMC often presents with systemic abnormalities (12). In this study, we found that the head-hip length and body mass index of MMC fetal mice were significantly lower than those of CRL fetal mice (Table 3) at each evaluated embryonic period, and the gap between the two groups became increasingly obvious as the pregnancy progressed.

**Table 3:** Summary of the weight and crown-rump length of fetuses between CRL and MMC.

	**Gestational age (days)**	**Numbers (*n*)**	**Crown-rump length (mm)**	**Weight (g)**
CRL	16	10	16.61 ± 1.04	0.63 ± 0.08
	18	14	24.78 ± 1.33	2.0 ± 0.16
	20	13	37.25 ± 2.78	4.14 ± 0.45
	22	14	44.72 ± 1.28	6.97 ± 0.52
MMC	16	11	14.42 ± 0.56^*^	0.45 ± 0.06^*^
	18	13	21.60 ± 0.68^*^	1.3 ± 0.1^*^
	20	12	31.6 ± 2.25^*^	3.37 ± 0.34^*^
	22	13	37.89 ± 3.12^*^	5.57 ± 0.86^*^

* *Values are expressed as the mean ± standard error of the mean*.

### Changes in Cell Proliferation and Apoptosis and Neuromuscular Development-Related mRNA in Fetal Rat Bladder Tissue at E16–E22

The development and differentiation of the rat bladder involves the expression of nerves and muscles and cell renewal in tissues. Our experiment use QPCR showed that the expression of proliferating cell nuclear antigen (PCNA) mRNA in bladder tissue gradually decreased from E16 to E22, and the change in PCNA mRNA expression in the MMC and CRL groups was relatively consistent, without a significant statistical difference (Figure 1C). Apoptosis plays an important role in the formation of tissues and organs. The results of the fluorescence qPCR analysis showed that there was no significant difference in the mRNA expression of cleaved caspase-3 between the MMC and CRL groups from E16 to E18; however, in the MMC group, apoptosis of bladder tissue was increased and significantly differed from that in the CRL group from E20 to E22 (Figure 1D). In summary, proliferation was inhibited in MMC rat fetuses at the early stage of bladder development (E16), and the expression of apoptosis-related genes was increased in the late stage of bladder development (E20–E22). In addition, bladder studies in children with MMC have shown that NB dysfunction in MMC is associated with abnormal bladder innervation. As shown in Figure 1E, with the progression of pregnancy, the expression of NeuN mRNA in normal embryo bladder increased with time, although the increase in NeuN mRNA in MMC bladder tissue slowed during E18–E22 and was significantly lower than that in the CRL group at E22. The expression of α-smooth muscle actin (α-SMA) mRNA in the MMC group was significantly lower than that in the CRL group at E22 (Figure 1F).

### Changes in Cell Proliferation, Apoptosis, and Neuromuscular Development-Related Proteins in Fetal Rat Bladder Tissue at E16–E22

**Figure 2:**
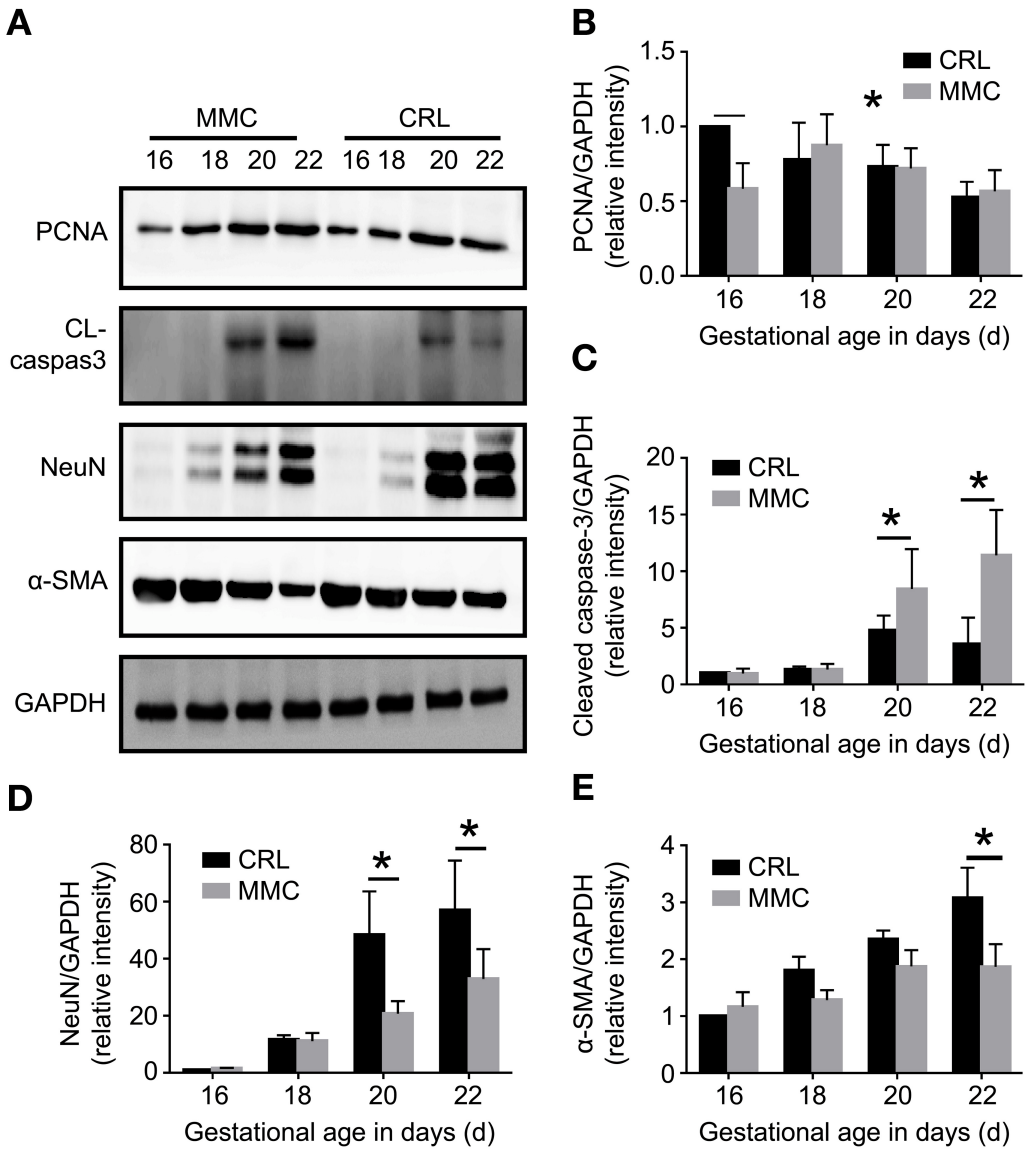
Cell proliferation, apoptosis, and neuromuscular development-related protein changes in fetal rat bladder at E16–E22. **(A)** Expression of PCNA, cleaved caspase-3, NeuN, and a-SMA proteins in the MMC and CRL groups at E16, E18, E20, and E22. **(B–E)** The expression of PCNA **(B)**, cleaved caspase-3 **(C)**, NeuN **(D)**, and α-SMA **(E)** in the MMC and CRL groups changed with the development of the embryos, *n* = 5, *P < 0.05. Data are expressed as means ± STDs. MMC, myelomeningocele; STD, standard deviation; PCNA, proliferating cell nuclear antigen; α-SMA, α-smooth muscle actin; NeuN, neuron-specific nuclear-binding protein; CRL, control group; CL-caspase3, cleaved caspase-3; GAPDH, glyceraldehyde-3-phosphate dehydrogenase.

Western blot results further verified the above results, as shown in Figure 2. The expression of PCNA protein in the CRL group reached its highest at E16, and then gradually decreased. The expression of PCNA protein was significantly lower in the MMC group than in the CRL group at E16, but did not significantly differ between the groups from E18 to E22 (Figure 2B). From E16 to E18, cleaved caspase-3 was not expressed, or was rarely expressed, in the MMC and CRL groups. The expression of cleaved caspase-3 in the bladder tissue of advanced embryos (E20–E22) was significantly higher in the MMC group than in the CRL group (Figure 2C). In addition, NeuN protein expression in the MMC and CRL groups increased with time, with no significant difference between the MMC and CRL groups from E16 to E18; however, the expression of NeuN protein was significantly lower in the MMC group than in the CRL group from E20 to E22 (Figure 2D). The protein expression of α-smooth was significantly lower in the MMC group than in the CRL group at E22 (Figure 2E), which was the same result as that in the qPCR experiment. Based on the qPCR and western blotting results, the neuromuscular development index of the bladder was still within the normal range in the early stage of embryonic development, although the expression of mRNA and protein was significantly decreased in the late stage of embryonic development, which may be related to multiple urinary system dysfunction after birth.

### Expression and Distribution of Bladder Cleaved Caspase-3 at E22

**Figure 3:**
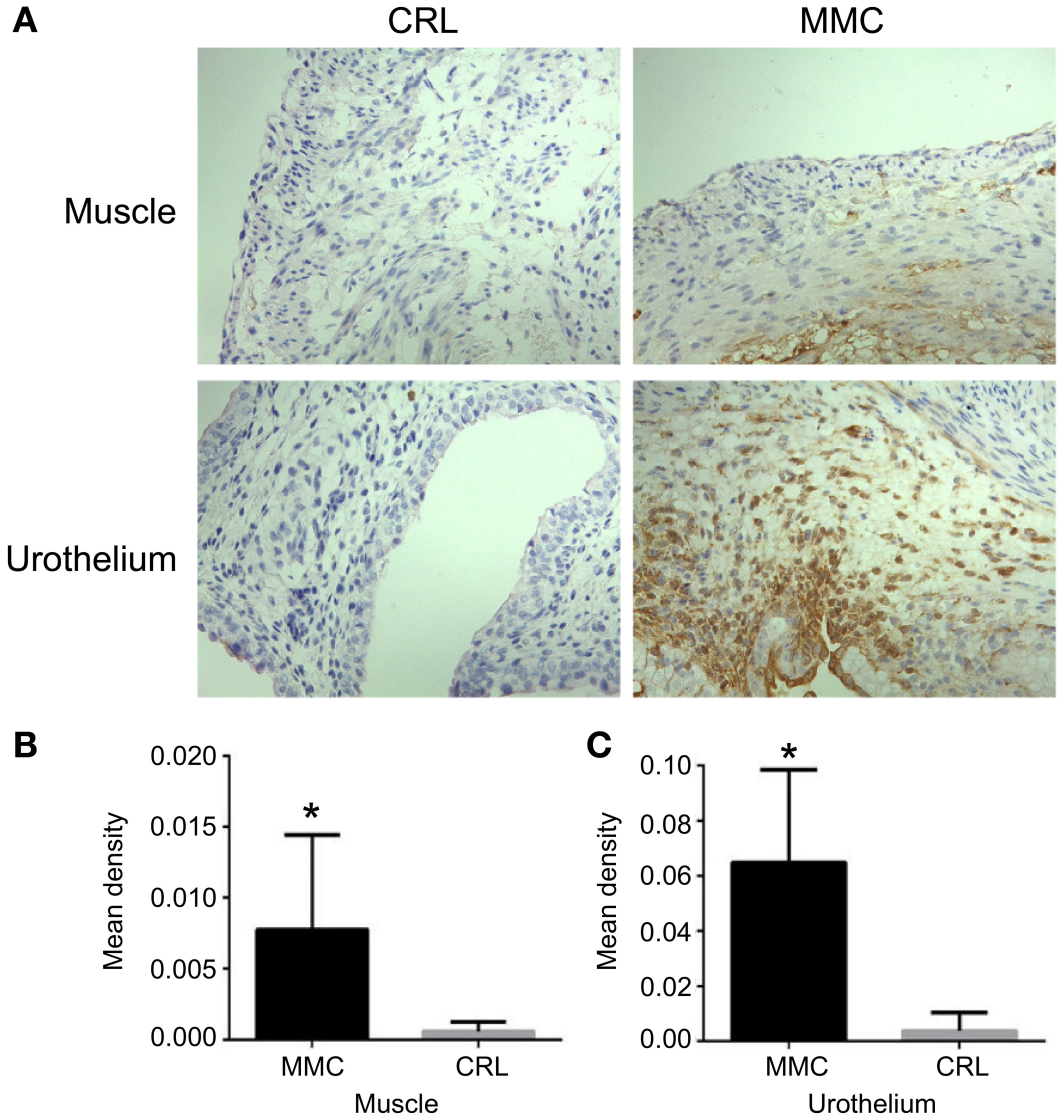
Expression and distribution of bladder cleaved caspase-3 at E22. **(A)** The distribution of cleaved caspase-3 at E22. **(B)** The expression of cleaved caspase-3 in the bladder muscle layer was significantly lower in the CRL group than in the MMC group. **(C)** The expression of cleaved caspase-3 in the bladder mucosa layer was significantly higher in the MMC group than in the CRL group, *n* = 6, *P < 0.05. Data are expressed as means ± STDs. MMC, myelomeningocele; STD, standard deviation; CRL, control.

Cleaved caspase-3 is a key molecule involved in cell apoptosis. Immunohistochemistry showed that the expression of cleaved caspase-3 in muscle or urothelium was significantly higher in the MMC group than in the CRL group, and the expression of cleaved caspase-3 was mainly concentrated in the bladder mucosa (Figure 3A). The expression of cleaved caspase-3 in the bladder muscle or urothelium was significantly higher in the MMC group than in the CRL group (Figures 3B,C). Inflammatory invasion and increased apoptosis of bladder mucosa may be a major inducer of bladder mucosa fibrosis after birth.

## Discussion

MMC is a common neural tube malformation, and 95% of children with MMC have NB. Early NB shows detrusor sphincter dyscoordination, increased residual urine in the bladder, increased bladder pressure, and bladder ureteral reflux, which causes repeated upper urinary tract infection and progressive renal scar formation (4, 13), late bladder smooth muscle hyperplasia, and trabecular formation, further increasing bladder pressure and aggravating renal damage. Urinary tract injury is a common complication of NB caused by MMC (4). Clinically, 58% of children with MMC show progressive deterioration of renal function within 3 years of birth without regular treatment (4, 14). However, the physiological and pathological development of NB remains controversial, and the lack of this understanding hinders the development of MMC-related treatment strategies for NB. Retinoic acid has been shown to be highly effective in inducing isolated MMC-like defects in fetal rats. Danzer et al. (9) show that exposure to 60 mg/kg RA at E10 (time of posterior neuropore closure in rats) leads to a pathology that has striking morphologic and clinical similarity to human MMC. We used 60 mg/kg RA at E10 not only to induce MMC fetal rat, but also it will not cause bladder dysfunction (9). Therefore, this prenatal rat model of RA exposure has been used to study both prenatal MMC symptoms and mechanisms of action, and thus has been helpful in understanding more about what may be occurring when spina bifida occurs in bladder.

The development and differentiation of the rat bladder is a gradual process, from organ formation to postpartum maturation, accompanied by the growth of the bladder. We observed that the expression of PCNA in the bladder tissue of normal fetal rats decreased gradually from E16 to E22. Smeulders (15) reported that the expression of PCNA in the bladder tissue of fetal rats decreased gradually from E14 to postnatal day 6. However, the expression of PCNA in the MMC group was significantly lower than that in the CRL group at E16 (*P* < 0.05), reached its peak at E18, and then gradually decreased, indicating that the proliferation of bladder cells in the MMC group was inhibited on E16. Wang et al. (16) reported that the cleft palate of the offspring of pregnant rats exposed to retinoic acid can inhibit the extension of the skeleton by inhibiting the proliferation of cells, which indicates that the occurrence of malformation in offspring is closely related to early tissue proliferation disorder. Lima TS (17) reported that the expression of apoptotic protein-related proteins was inhibited by cytoplasmic expression of PCNA, thereby prolonging cell lifespan. In the present experiment, we found that some MMC fetal rats had a small bladder. The expression of PCNA in MMC fetal mice was decreased at E16, a critical period of organ formation, which may lead to the absence of the bladder or occurrence of a small bladder. Follow-up experiments are needed to determine whether the small bladder is caused by the inhibition of proliferation.

Apoptosis plays an important role in the formation of tissues and organs. In this study, the expression of cleaved caspase-3 was not detected in MMC and CRL groups at E16 and E18. However, the expression of cleaved caspase-3 was significantly higher in the MMC group than in the CRL group at E20 and E22. Further, we found that the expression of cleaved caspase-3 in the muscle and mucosal layers of the bladder was higher in the MMC group than in the CRL group, which may be related to the apoptosis of smooth muscle cells, involved in the remodeling of bladder, in late embryo. Nomiya et al. (18) showed that oxidative stress and inflammation induced apoptosis may be key factors in the development of bladder overactivity in chronic bladder ischemia. Similarly, oxidative damage caused by ovariectomy has been shown to result in enhanced urination frequency and reduced bladder compliance, and bladder function can be restored through anti-inflammatory and antioxidant properties (18, 19). As the expression of cleaved caspase-3 in the bladder mucosa and muscle was significantly higher in the MMC group than in the CRL group at E22, an increase in inflammatory mediators and apoptotic proteins in the bladder mucosa and muscle in late embryos may be a major cause of bladder dysfunction in MMC.

Bladder studies in children with MMC have shown that NB dysfunction in MMC is associated with abnormal innervation of the bladder. Shapiro et al. (20) reported that the density of neurons in the bladder wall is decreased in fetuses with MMC. Gup et al. (14) found that the density of muscarinic cholinergic receptors in the bladder is significantly decreased in children with MMC. Haferkamp et al. (21) recently analyzed the ultrastructural changes of abnormal bladder detrusor innervation in adolescents with MMC, and showed that NB dysfunction is not only associated with axonal degeneration and depletion of terminal organelles (such as vesicles and mitochondria), but also with increased width of the neuromuscular junction, suggesting that children with MMC-related NB have neurogenic developmental abnormalities. In this study, the expression of NeuN in the bladder of MMC fetal rats was also significantly downregulated in the late embryonic stage (E20 and E22), further confirming that abnormal nerve distribution existed in the late embryonic stage of MMC fetal rats, and that bladder dysfunction after birth may be related to abnormal nerve distribution at an early stage. It has been confirmed that neural function damage in MMC fetuses shows a clear time difference. In the early embryotic development, MMC fetuses show normal lower limb motor function, while in the late embryotic development, the neural function deteriorates progressively. In this study, we also observed that there were no significant differences in the NeuN protein expression between the groups at E16 and E18. This indicated normal neurodevelopment of the bladder during early embryotic development (E16 and E18). The observed neurodevelopmental malformation in the late embryotic development was consistent with the reported loss of peripheral limb function. From another perspective, we believe that there is a correlation between nerve development and spinal cord injury in fetal rats with MMC.

The change in bladder morphology is not caused by urinary tract infection or detrusor sphincter dyscoordination in later stages. Changes in bladder morphology occur during the embryonic and neonatal stages. qPCR and western blot results showed that the expression of α-SMA was gradually upregulated in the MMC and CRL groups; however, the expression of α-SMA at E22 was significantly lower in the MMC group than in the CRL group. Although Danzer et al. (9) reported that the expression of α-SMA at E22 in MMC bladders was not significantly different from that in the normal bladders, the muscle tension of MMC bladders decreased significantly at E22; this indicated developmental malformation of bladder muscle contraction in the late embryonic stage. Complete bladder innervation may be a prerequisite for the morphogenesis and development of bladder smooth muscle. It was also found that the expression of NeuN was significantly downregulated at E20, indicating that neurodevelopmental malformations may lead to secondary atrophy of local bladder smooth muscle due to denervation.

A limitation of this study is that it did not explore the bladder function changes and molecular changes secondary to spinal cord injuries in fetal rats with MMC simultaneously. It also did not investigate whether these changes were accompanied by multiple system malformation. We greatly consider investigating myelomeningocele and bladder developmental malformations as worthwhile. However, in the early phases of our research program, spinal cord repair in fetal mouse embryos with MMC was conducted on E17, and we did not find any significant improvement in bladder molecular changes in later stages. This suggests that, to a certain extent, even if fetal surgery is carried out in the future to repair the spinal cord of fetuses with MMC, the congenital bladder development malformation should not be ignored. Therefore, it is necessary to address the fetal bladder dysfunction in MMC. There is still a long way to go to treat this disease.

## Conclusions

In conclusion, the development of the bladder in rats with MMC is not due to a single neurodegeneration, and the failure of neural tube closure does not affect the innervation of peripheral tissues and organs in the early stage. Degeneration of nerve function mostly occurs during late embryonic development. The occurrence of NB is accompanied by abnormal early proliferation. Inflammation of the bladder epithelium in the late embryonic stage mediates the apoptosis of tissue cells, which may be involved in the remodeling of the bladder tissue in the late embryonic stage. The occurrence of bladder dysfunction after birth indicates a correlation between these two factors. The inhibition of proliferation, promotion of apoptosis, and reduction of the bladder nerve- and smooth muscle-related protein synthesis were characteristic of bladder development in fetal rats with MMC, which established the bladder dysfunction after birth.

## Data Availability Statement

The original contributions presented in the study are included in the article/supplementary material, further inquiries can be directed to the corresponding authors.

## Ethics Statement

The animal study was reviewed and approved by the Institutional Review Board of the Children's Hospital of Fudan University.

## Author Contributions

YL contributed to the study design, data collection, data analysis, and drafted the manuscript. LC contributed to the animal experiment, data collection, data analysis, interpretation, and wrote the manuscript. YB contributed to the study design. JS and HC contributed to the animal experiment. YM contributed to data collection. All authors contributed to the article and approved the submitted version.

## Conflict of Interest

The authors declare that the research was conducted in the absence of any commercial or financial relationships that could be construed as a potential conflict of interest.

## Publisher's Note

All claims expressed in this article are solely those of the authors and do not necessarily represent those of their affiliated organizations, or those of the publisher, the editors and the reviewers. Any product that may be evaluated in this article, or claim that may be made by its manufacturer, is not guaranteed or endorsed by the publisher.
